# Racial and Ethnic Differences in Health Behaviors and Preventive Health Services Among Prostate Cancer Survivors in the United States

**DOI:** 10.5888/pcd13.160148

**Published:** 2016-07-21

**Authors:** Jun Li, Trevor D. Thompson, Thomas B. Richards, C. Brooke Steele

**Affiliations:** Author Affiliations: Trevor D. Thompson, Thomas B. Richards, C. Brooke Steele, Division of Cancer Prevention and Control, National Center for Chronic Disease Prevention and Health Promotion, Atlanta, Georgia.

## Abstract

**Introduction:**

Little is known about how health behaviors and receipt of preventive health care differ by race and ethnicity among prostate cancer survivors. The purpose of this study was to identify differences in the prevalence of 7 modifiable factors related to prostate cancer: smoking, alcohol consumption, physical inactivity, weight, colorectal cancer screening, influenza vaccination, and pneumococcal vaccination.

**Methods:**

We used data from the 2010 Behavioral Risk Factor Surveillance System to calculate the racial/ethnic prevalence of sociodemographic and health-related characteristics, health behaviors, and preventive health care among prostate cancer survivors. Adjusted prevalence estimates were calculated by using multivariable logistic regression.

**Results:**

We identified 8,016 men with a history of prostate cancer. Multivariable analyses indicated that more black men reported being obese (29.9%; 95% confidence interval [CI], 24.5%–35.9%) than white men (22.8%; 95% CI, 21.1%–24.6%). More white men (3.6%; 95% CI, 2.9%–4.5%) reported consuming more than 2 alcoholic drinks per day than black men (0.9%; 95% CI, 0.4%–2.0%). More white men aged 65 or older reported receiving pneumococcal vaccine (74.2%; 95% CI, 72.2%–76.1%) than black men of the same age (63.2%; 95% CI, 54.8%–70.8%).We did not observe any differences in the prevalence of health behaviors and preventive health care between white men and men in Hispanic or other race categories.

**Conclusion:**

Differences in alcohol consumption, obesity, and receipt of pneumococcal vaccination existed only between black and white prostate cancer survivors. These differences underscore the need to develop culturally appropriate, evidence-based interventions to reduce excessive alcohol consumption, maintain a healthy weight, and promote pneumococcal vaccination among prostate cancer survivors.

## Introduction

Because of widespread prostate cancer screening, advances in cancer diagnosis and treatment, and the slow-growing nature of most prostate cancers, cancer survival rates have improved substantially (1). By the end of 2011, the number of prostate cancer survivors approached 2.8 million and accounted for 44% of all US male cancer survivors (2). The number of prostate cancer survivors is expected to increase over time because of population aging (2).

Studies have consistently shown that unhealthy behaviors, such as poor diet, physical inactivity, and cigarette smoking, increase the risk of prostate cancer recurrence, secondary cancers, other chronic diseases, and death (3). Prostate cancer survivors are more likely to die of comorbid diseases than of prostate cancer (4). Thus, attention to health behaviors and preventive health care is an important aspect of quality care for prostate cancer survivors. In 2014, the American Cancer Society released guidelines for care of prostate cancer survivors to help survivors achieve optimal health and quality of life (3).

Although racial and ethnic differences in health behaviors and preventive health care have been well-documented among the general population (5), patterns may change after cancer diagnosis. To our knowledge, racial/ethnic disparities have been studied among breast cancer and colorectal cancer (CRC) cancer survivors but not prostate cancer survivors (6). We assessed racial and ethnic differences in the prevalence of 7 risk factors — smoking, alcohol consumption, physical inactivity, obesity, CRC screening, influenza vaccination, and pneumococcal vaccination — that could indicate opportunities for prevention of comorbid diseases and death among prostate cancer survivors. Cancer control planners could use this information to tailor and prioritize public health interventions by race and ethnicity to improve the health and well-being of survivors.

## Methods

The Behavioral Risk Factor Surveillance System (BRFSS) is an ongoing, state-based, random-digit–dialed telephone survey of the noninstitutionalized US civilian population aged 18 years or older (http://www.cdc.gov/BRFSS/). The BRFSS collects information about diseases, health-related behaviors, preventive health practices, and access to health care. We analyzed 2010 BRFSS data from all 50 states and the District of Columbia. The median response rate was 54.6%, and the median cooperation rate was 76.9% (7). Because this study analyzed secondary data from a publicly accessible dataset, it did not require institutional review board approval.

To identify prostate cancer survivors, we used the question, “Have you ever been told by a doctor, nurse, or other health professional that you had prostate cancer?” We restricted our analysis to men aged 40 years or older who reported a history of prostate cancer, because BRFSS did not collect prostate cancer information among men younger than 40. We analyzed 4 racial and ethnic categories: non-Hispanic white (hereafter, “white”), non-Hispanic black (hereafter, “black”), non-Hispanic other (includes Asian and Pacific Islanders, American Indians and Alaska Natives, and others; hereafter “other race”), and Hispanic.

We examined the following factors: demographics (age, education level, employment status, and marital status), health care access (health insurance status, medical cost concerns, having a usual health care provider, and time since last checkup), and emotional support. We classified emotional support into a 3-level variable: always or usually, sometimes, and rarely or never in response to the question, “How often do you get the social and emotional support you need?” We also identified chronic conditions, such as a history of diabetes (all types), myocardial infarction, heart disease, stroke, or current asthma. The status of these chronic conditions were classified as a yes/no variable. We grouped the number of chronic conditions into 4 categories: 0, 1, 2, and 3 or more. We defined disability status as a yes/no variable on the basis of the question, “Are you limited in any way in any activities because of physical, mental, and emotional problems?”

We defined obesity as having a body mass index (BMI) of 30 kg/m^2^ or higher. We dichotomized alcohol consumption into more than 2 drinks per day or 2 drinks per day or less on the basis of American Cancer Society recommendations for alcohol consumption among prostate cancer survivors (3). We classified physical inactivity status with yes or no answers to the question, “During the past month, other than your regular job, did you participate in any physical activities or exercise, such as running, calisthenics, golf, gardening, or walking for exercise?” We identified current cigarette smokers as adults who had smoked at least 100 cigarettes during their lifetime and were smoking at the time the survey was conducted.

On the basis of national vaccination recommendations and cancer screening recommendations (8–10), we included 3 preventive health service measures for age-eligible persons: up-to-date influenza vaccination, pneumococcal vaccination for men aged 65 years or older, and CRC screening for men aged 50 to 75 years. We determined the status of up-to-date influenza and pneumococcal vaccinations with the following survey questions: “During the past 12 months, have you had a seasonal flu shot?”; “During the past 12 months, have you had a seasonal flu vaccine that was sprayed in your nose?”; and “Have you ever had a pneumonia shot?” CRC screening was assessed according to US Preventive Services Task Force recommendations as receipt of any of the following: fecal occult blood test within 1 year, sigmoidoscopy within 5 years and fecal occult blood test within 3 years, or colonoscopy within 10 years (9).

We calculated weighted percentage estimates with 95% confidence intervals (CIs) for sociodemographic and health-related characteristics, health behaviors, and use of preventive health care, stratified by racial and ethnic group. Differences by racial and ethnic group were assessed by using the Pearson Wald F test. A multivariable logistic regression model was constructed to examine racial and ethnic differences in health behaviors and preventive health care use after adjustment for sociodemographic and health-related characteristics. We present the adjusted associations of race and ethnicity with health behaviors and preventive health care as percentages by using predictive margins. The predictive margin for a specific group represents the average predicted response if everyone in the sample had been in that group (11). *P* values for pairwise comparisons between white and other racial and ethnic groups were based on the Wald F test. We analyzed all data by using SAS, version 9.3 (SAS Institute, Inc) with SUDAAN, version 11.0.0 (RTI International) to account for complex sampling design and to allow for weighted estimates. *P* values less than or equal to .05 were deemed significant.

## Results

Of 8,016 men interviewed for the 2010 BRFSS who reported a history of prostate cancer, most were white (79.7%), followed by black (11.2%), Hispanic (5.8%), and other race (3.3%). All sociodemographic and health-related characteristics for prostate cancer survivors varied significantly by race and ethnicity (*P* ≤.05), except having a usual health care provider (Table 1). Prevalence rates of the following categories were highest among white men: being aged 65 years or older, having health insurance, having fewer medical cost concerns, having a usual health care provider, and always having emotional support. White men (6.2%) had a lower prevalence of unemployment than black men (11.1%) and Hispanic men (18.8%). Men classified as other race had a higher prevalence of having a college degree and being married or living together than black men and Hispanic men. Hispanic men had a higher prevalence of having less than a high school education (40.1%) than white men (7.0%) and black men (21.6%).

**Table 1:** Sociodemographic and Health-Related Characteristics Among Men Aged 40 Years or Older (N = 8,016^a^) With a History of Prostate Cancer, United States, BRFSS 2010

Characteristic	White, % (95% CI)	Black, % (95% CI)	Other Race, % (95% CI)	Hispanic, % (95% CI)	*P* Value
**Age, y**	n = 6,886	n = 673	n = 205	n = 226	<.001
40–64	23.0 (21.2–24.9)	34.5 (28.7–40.9)	39.4 (26.2–54.3)	38.5 (27.8–50.4)	
≥65	77.0 (75.1–78.8)	65.5 (59.1–71.3)	60.6 (45.7–73.8)	61.5 (49.6–72.2)	
**Education level**	n = 6,894	n = 674	n = 203	n = 224	<.001
<High school	7.0 (6.1–8.0)	21.6 (16.5–27.6)	15.9 (7.2–31.7)	40.1 (29.2–52.2)	
High school graduate	25.8 (24.2–27.5)	32.1 (26.5–38.3)	17.2 (10.5–26.9)	20.3 (12.9–30.4)	
Some college	22.9 (21.3–24.7)	22.6 (18.0–28.0)	15.7 (8.8–26.6)	15.3 (9.8–23.0)	
College graduate	44.2 (42.3–46.1)	23.7 (18.9–29.3)	51.1 (37.1–65.0)	24.3 (15.2–36.6)	
**Employment status**	n = 6,887	n = 674	n = 204	n = 225	.006
Employed	24.8 (23.0–26.7)	23.5 (18.4–29.5)	34.9 (21.4–51.3)	24.1 (15.2–35.9)	
Not employed	6.2 (5.3–7.3)	11.1 (8.3–14.6)	9.6 (5.2–16.9)	18.8 (11.2–29.9)	
Retired	69.0 (67.1–70.9)	65.5 (59.3–71.1)	55.6 (40.8–69.4)	57.2 (45.2–68.3)	
**Marital status**	n = 6,902	n = 675	n = 203	n = 225	<.001
Married or living together	77.8 (76.3–79.2)	66.3 (60.8–71.4)	82.0 (72.8–88.5)	57.9 (45.7–69.2)	
Divorced, separated, or widowed	18.8 (17.5–20.2)	28.0 (23.3–33.2)	16.5 (10.2–25.5)	32.0 (22.0–44.1)	
Never married	3.4 (2.8–4.0)	5.7 (3.9–8.3)	1.6 (0.7–3.3)	10.1 (3.9–23.6)	
**Health insurance**	n = 6,900	n = 675	n = 204	n = 226	.046
Yes	97.9 (97.1–98.4)	94.6 (90.7–97.0)	88.8 (70.6–96.3)	92.1 (80.6–97.0)	
No	2.1 (1.6–2.9)	5.4 (3.0–9.3)	11.2 (3.7–29.4)	7.9 (3.0–19.4)	
**Time since last checkup**	n = 6,843	n = 668	n = 199	n = 223	<.001
Within past year	86.9 (85.5–88.1)	90.3 (85.6–93.6)	85.9 (74.9–92.6)	81.6 (68.9–89.9)	
≤2 years	7.3 (6.4– 8.4)	6.1 (3.5–10.3)	5.6 (2.3–12.9)	2.3 (0.9–5.3)	
2–<5 years	2.8 (2.3–3.5)	2.7 (1.3–5.8)	1.8 (0.6–5.1)	9.2 (3.2–23.7)	
≥5 years	2.7 (2.1–3.3)	0.9 (0.3–3.1)	6.1 (1.8–18.2)	3.5 (1.2–9.6)	
Never	0.3 (0.2–0.5)	0.0 (.–.)	0.6 (0.1–2.4)	3.4 (0.8–13.3)	
**Medical cost concerns**	n = 6,896	n = 672	n = 205	n = 224	.001
Yes	4.2 (3.4–5.0)	8.7 (6.1–12.4)	21.3 (10.5–38.4)	11.7 (6.3–20.7)	
No	95.8 (95.0–96.6)	91.3 (87.6–93.9)	78.7 (61.6–89.5)	88.3 (79.3–93.7)	
**Has usual health care provider**	n = 6,894	n = 671	n = 205	n = 224	.12
Yes	96.6 (95.9–97.3)	93.6 (89.5–96.2)	95.4 (86.2–98.6)	91.5 (83.0–95.9)	
No	3.4 (2.7–4.1)	6.4 (3.8–10.5)	4.6 (1.4–13.8)	8.5 (4.1–17.0)	
**Emotional support**	n = 6,614	n = 636	n = 194	n = 213	<.001
Always/usually	81.5 (80.0–82.9)	68.7 (62.5–74.2)	63.0 (48.3–75.6)	61.9 (49.4–72.9)	
Sometimes	6.9 (6.0–7.9)	15.8 (11.8–20.9)	15.0 (6.8–30.0)	19.1 (10.8–31.5)	
Rare/none	11.7 (10.5–12.9)	15.5 (11.5–20.7)	22.0 (12.7–35.3)	19.1 (11.3–30.4)	
**Has chronic conditions^b^**	n = 6,640	n = 648	n = 191	n = 219	<.001
0	58.5 (56.5–60.3)	43.4 (37.4–49.6)	47.5 (33.3–62.1)	62.2 (50.9–72.2)	
1	24.6 (23.0–26.3)	37.1 (30.8–43.9)	24.3 (15.2–36.7)	25.5 (17.0–36.3)	
2	11.6 (10.4–12.9)	15.9 (11.9–21.0)	10.1 (4.3–21.8)	5.0 (2.5–9.8)	
≥3	5.3 (4.5–6.3)	3.5 (2.0–5.9)	18.1 (8.2–35.4)	7.4 (3.7–14.3)	
**Disability**	n = 6,873	n = 668	n = 203	n = 226	.02
Yes	36.0 (34.2–37.9)	30.6 (25.0–36.8)	24.8 (14.3–39.4)	25.2 (17.2–35.4)	
No	64.0 (62.1–65.8)	69.4 (63.2–75.0)	75.2 (60.6–85.7)	74.8 (64.6–82.8)	

Abbreviations: BRFSS, Behavioral Risk Factor Surveillance System; CI, confidence interval.

a Variations of total count by variable might be caused by “don't know,” refused, or missing responses.

b Chronic conditions included are diabetes, myocardial infarction, heart disease, and current asthma.

Overall, there were significant racial and ethnic differences in histories of diabetes, total number of chronic conditions, and disability status (*P* ≤.05). Black men had a higher prevalence of diabetes (33.7%) than white (18.6%) and Hispanic men (19.5%) (data not shown). Men of other race had a higher prevalence of having 3 or more chronic conditions (18.1%) than black men (3.5%) and white men (5.3%). White men had a higher percentage of disability (36.0%) than Hispanic men (25.2%).

Significant racial or ethnic differences were observed in current cigarette smoking status, consumption of more than 2 alcoholic drinks per day, obesity, and receipt of influenza and pneumococcal vaccines (*P* ≤.05) (Table 2). Black men had a higher prevalence of being current cigarette smokers (15.1%) than white men (8.4%). Black men also reported a higher percentage of obesity (33.2%) than white men (22.7%). Hispanic men (7.0%) and white men (3.7%) reported higher percentages of consuming more than 2 drinks per day than did black men (0.8%).

**Table 2:** Weighted Percentages for Selected Health Behaviors and Preventive Health Care Among Men Aged 40 Years or Older (N = 8,016^a^) With a History of Prostate Cancer, United States, BRFSS 2010

Characteristic	White % (95% CI)	Black % (95% CI)	Other Race % (95% CI)	Hispanic % (95% CI)	*P* Value
**Current cigarette smoker^b^**	n = 6,867	n = 671	n = 204	n = 222	.005
Yes	8.4 (7.3- 9.6)	15.1 (11.7–19.3)	8.0 (3.7–16.7)	15.0 (7.8–26.9)	
No	91.6 (90.4–92.7)	84.9 (80.7–88.3)	92.0 (83.3–96.3)	85.0 (73.1–92.2)	
**Drink >2 alcoholic beverages/d**	N = 6,767	N = 657	N = 201	N = 218	<.001
Yes	3.7 (3.0- 4.4)	0.8 (0.4- 1.8)	1.7 (0.5- 5.5)	7.0 (2.7–17.4)	
No	96.3 (95.6–97.0)	99.2 (98.2–99.6)	98.3 (94.5–99.5)	93.0 (82.6–97.3)	
**Physically inactive^c^**	N = 6,900	N = 673	N = 203	N = 224	.13
Yes	24.8 (23.2–26.4)	31.1 (25.8–36.9)	32.7 (20.6–47.5)	26.9 (17.3–39.4)	
No	75.2 (73.6–76.8)	68.9 (63.1–74.2)	67.3 (52.5–79.4)	73.1 (60.6–82.7)	
**Body mass index, kg/m^2^**	N = 6,873	N = 662	N = 204	N = 224	.007
<25.0	28.7 (27.0–30.5)	27.4 (21.8–34.0)	39.2 (26.7–53.4)	30.8 (20.6–43.3)	
25.0–29.9	48.6 (46.7–50.5)	39.3 (33.5–45.5)	34.5 (22.7–48.6)	44.3 (33.4–55.9)	
≥30.0	22.7 (21.1–24.3)	33.2 (27.7–39.2)	26.3 (14.9–42.0)	24.9 (16.2–36.2)	
**Had influenza vaccination during the preceding 12 months**	N = 6,870	N = 674	N = 203	N = 226	.01
Yes	70.3 (68.4–72.0)	63.7 (57.8–69.2)	58.5 (43.9–71.8)	58.0 (46.2–68.9)	
No	29.7 (28.0–31.6)	36.3 (30.8–42.2)	41.5 (28.2–56.1)	42.0 (31.1–53.8)	
**Had pneumococcal vaccination (if ≥65 y)**	N = 5407	N = 451	N = 141	N = 154	<.001
Yes	74.6 (72.7–76.4)	62.1 (54.5–69.2)	60.2 (41.4–76.4)	50.9 (35.9–65.8)	
No	25.4 (23.6–27.3)	37.9 (30.8–45.5)	39.8 (23.6–58.6)	49.1 (34.2–64.1)	
**Screened for colorectal cancer (if 50–75 y)^d^**	N = 3541	N = 426	N = 104	N = 133	.08
Yes	84.8 (82.7–86.6)	77.8 (70.6–83.7)	66.0 (42.9–83.4)	77.9 (61.7–88.5)	
No	15.2 (13.4–17.3)	22.2 (16.3–29.4)	34.0 (16.6–57.1)	22.1 (11.5–38.3)	

Abbreviations: BRFSS, Behavioral Risk Factor Surveillance System; CI, confidence interval.

a Variations of total count by variable might be caused by “don’t know,” refused, or missing responses.

b Defined as having smoked 100 cigarettes in lifetime and currently smoking.

c Defined by a no response to the question, “During the past month, other than your regular job, did participate in any physical activities or exercise, such as running, calisthenics, golf, gardening, or walking for exercise?”

d Screened according to US Preventive Services Task Force recommendations (fecal occult blood test within 1 year, sigmoidoscopy within 5 years and fecal occult blood test within 3 years, or colonoscopy within 10 years).

After adjusting for sociodemographic and health-related characteristics, multivariable logistic regression analyses (Table 3) indicated that racial/ethnic differences remained significant for consuming more than 2 alcoholic drinks per day, obesity, and receipt of pneumococcal vaccine. More black men reported being obese (29.9%; 95% CI, 24.5%–35.9%) than white men (22.8%; 95% CI, 21.1%–24.6%). More white men (3.6%; 95% CI, 2.9%–4.5%) reported consuming more than 2 alcoholic drinks per day than black men (0.9%; 95% CI, 0.4%–2.0%). More white men aged 65 or older reported receiving pneumococcal vaccine (74.2%; 95% CI, 72.2%–76.1%) than black men of the same age (63.2%; 95% CI, 54.8%–70.8%). We did not observe significant differences in the prevalence of health behaviors and receipt of preventive health care between white men and men of Hispanic or other race.

**Table 3:** Multivariate Adjusted Percentages^a^ of Selected Health Behaviors and Preventive Health Care Among Men Aged 40 Years or Older With a History of Prostate Cancer, United States, BRFSS 2010

Characteristic	% (95% CI)	*P* Value^b^
**Current cigarette smoker^c^**		
White	9.3 (8.1–10.8)	NA
Black	11.2 (8.0–15.4)	.37
Other race	7.4 (4.0–13.5)	.43
Hispanic	8.9 (5.3–14.7)	.87
**Drink >2 alcoholic beverages/d**		
White	3.6 (2.9–4.5)	NA
Black	0.9 (0.4- 2.0)	<.001
Other race	2.1 (0.6- 6.9)	.28
Hispanic	7.2 (3.2–15.4)	.22
**Physically inactive^d^**		
White	24.6 (22.9–26.4)	NA
Black	27.1 (21.9–32.9)	.41
Other race	27.9 (18.3–40.2)	.56
Hispanic	22.5 (15.2–32.0)	.63
**Obese (≥30.0 kg/m^2^)**		
White	22.8 (21.1–24.6)	NA
Black	29.9 (24.5–35.9)	.02
Other race	23.4 (13.1–38.4)	.93
Hispanic	22.8 (15.4–32.5)	.99
**Had influenza vaccination during the preceding 12 months**		
White	69.8 (67.9–71.8)	NA
Black	65.4 (59.1–71.2)	.18
Other race	66.1 (51.9–77.9)	.58
Hispanic	61.6 (50.0–72.0)	.15
**Had pneumococcal vaccination (if ≥65 y)**		
White	74.2 (72.2–76.1)	NA
Black	63.2 (54.8–70.8)	.009
Other race	61.9 (43.1–77.7)	.18
Hispanic	59.8 (41.9–75.4)	.11
**Screened for colorectal cancer (if 50–75 y)^e^**		
White	82.5 (80.7–84.1)	NA
Black	81.2 (76.1–85.5)	.62
Other race	74.9 (52.4–89.0)	.43
Hispanic	81.6 (72.5–88.2)	.83

Abbreviations: BRFSS: Behavioral Risk Factor Surveillance System; CI: confidence interval; NA, not applicable.

a Adjusted for age, education, employment, marital status, health insurance, time since last checkup, medical cost concern, usual health care provider, emotional support, diabetes, myocardial infarction, heart disease, asthma, and disabilities.

b *P* values for pairwise comparisons with whites are based on Wald F statistics.

c Defined as having smoked 100 cigarettes in lifetime and currently smoking.

d Defined by a no response to the question, “During the past month, other than your regular job, did participate in any physical activities or exercise, such as running, calisthenics, golf, gardening, or walking for exercise?”

e Screened according to US Preventive Services Task Force recommendations (fecal occult blood test within 1 year, sigmoidoscopy within 5 years and fecal occult blood test within 3 years, or colonoscopy within 10 years).

## Discussion

By using the BRFSS, the largest telephone survey of prostate cancer survivors in the United States, we analyzed whether racial and ethnic differences existed in 7 potential risk factors after prostate cancer diagnoses. Although racial and ethnic differences were not observed for physical inactivity, smoking, CRC screening, and influenza vaccination, we found that more white prostate cancer survivors reported drinking alcohol and receiving a pneumococcal vaccination than black survivors; more black survivors reported being obese than white survivors.

These differences among black and white populations have been corroborated by previous studies focusing on the general US population and breast cancer survivors. For example, Kanny and colleagues analyzed 2011 BRFSS data and reported that black adults had a substantially lower prevalence of binge drinking (14.2%) than white adults (21.1%) (12). In addition, White and colleagues found that white breast cancer survivors were more likely to be heavy alcohol drinkers (4.3%), but less likely to be obese (23.7%) than black breast cancer survivors (0.9% and 32.3%, respectively) (13). Likewise, among men aged 20 years or older participating in a 2007–2008 national survey, prevalence of obesity was significantly higher among blacks (37.3%) than whites (31.9%) (14).

More than one-third of US adults are obese (15). Although the link between obesity and risk of prostate cancer is not well established, studies suggested associations between obesity and prostate cancer-specific mortality and biochemical recurrence detected via prostate-specific antigen test (16–19). In addition, a retrospective, multi-institutional pooled analysis suggested that increased BMI could partially account for the disparity in biochemical recurrence of prostate cancer among black and white men (20). Overweight and obesity are risk factors for many other chronic diseases and conditions, including heart disease, hypertension, and diabetes. Some of these conditions are common among prostate cancer survivors. In one study, 20.1% of survivors reported having heart disease, 58.3% reported hypertension, and 23.7% reported diabetes (21). Because of the high prevalence of obesity and its adverse effects on prostate cancer outcomes and other chronic conditions, the American Cancer Society suggests that primary care physicians routinely assess BMI among prostate cancer survivors across their cancer survivorship continuum and recommends that survivors maintain a healthy weight (3). Differences in prostate cancer mortality rates between black and white men was extensively studied in relationship to socioeconomic status, tumor characteristics, treatment method, and quality of care (22–25). However, the role that obesity may play in racial disparities in prostate cancer mortality was not well studied.

Excessive alcohol consumption led to about 75,000 deaths in the United States from 2006 through 2010, making it a leading cause of premature death (26); however, excessive alcohol consumption was not shown to increase the risk of prostate cancer (27). Associations between alcohol use and prostate cancer-specific deaths are inconclusive (28). Alcohol should be avoided among prostate cancer survivors undergoing chemotherapy or biological treatments because of an increased risk of side effects resulting from alcohol–medication interactions (27).

Studies showed that racial or ethnic minorities are less likely to use preventive health care than whites (29,30). However, we did not find any differences between blacks and whites for receipt of CRC screening in our study. The Advisory Committee on Immunization Practices recommends influenza and pneumococcal vaccines for adults who meet the eligibility criteria (8,^10^). We observed significant racial/ethnic variation for receipt of pneumococcal vaccine among prostate cancer survivors but not for receipt of influenza vaccine. Palmer and colleagues examined 2006–2010 National Health Interview Survey data for male cancer survivors and reported that among men aged 65 years or older, black men were twice as likely as white men not to receive influenza vaccine and pneumococcal vaccine (6). The discrepancy between our findings for receipt of influenza vaccine and the findings of Palmer and colleagues might be because our study populations were younger (≥40 years) than theirs (≥65 years).

Our study has several limitations. First, BRFSS data were self-reported, and the findings may be subject to recall and social desirability biases. Second, although our study used nationwide data, BRFSS samples only noninstitutionalized US citizens with landline telephones. Cancer survivors who lived in prisons, nursing homes, long-term care facilities, or hospice were excluded. Third, we could not determine when cancer was diagnosed or whether survivors were currently having symptoms of cancer or were currently being treated, because this information was not collected. Fourth, racial/ethnic disparities in health behaviors and preventive health care services may have been due to factors we did not investigate, such as daily servings of fruits and vegetables, the type of health care provider from whom the respondent received services (ie, from a primary care provider or from a specialist), treatments and treatment-related comorbidities, and structural barriers to adopting healthy behaviors. BRFSS does not collect this information. Fifth, the study was underpowered to examine prostate cancer differences for Hispanics and other race; future studies should include more Hispanics and other populations. Sixth, we might have overestimated CRC screening rates, because some reported tests could be used for diagnostic and surveillance purposes. Finally, given the low response rate of 54.6% in the 2010 survey, our population-based estimates might reflect selection bias. However, the calculations of probability weights, which were adjusted for differences in probability of selection and nonresponse, might have partially corrected for any bias.

In conclusion, obesity and excessive alcohol use are preventable causes of death and comorbidity among prostate cancer survivors in the United States. The preventive health care service measures included in this study (eg, vaccinations, CRC screening) are evidence-based and cost-effective (8–10). Our study identified only significant differences in obesity, alcohol consumption, and pneumococcal vaccinations for black and white men, underscoring the need to develop culturally appropriate, evidence-based interventions to reduce excessive alcohol consumption, maintain a healthy weight, and promote pneumococcal vaccinations among prostate cancer survivors.
